# Supplementary material for: Modeling glioblastoma heterogeneity as a dynamic network of cell states
Source: Mol Syst Biol. 2021 Sep 16;17(9):e10105. doi: 10.15252/msb.202010105 (PMC8444284; doi:10.15252/msb.202010105)
Supplement: Supplementary file 6 — Source Data for Figure 5 [file MSB-17-e10105-s004.zip › Figure5A_sourcedata/GSEA_3017/nosigdb_stateB.GseaPreranked.1622795021725/RICHARDS_NOWAKOWSKI_SCIENCE_2017_IN_CTX_CGE1_UPREG.html]

Details for gene set RICHARDS\_NOWAKOWSKI\_SCIENCE\_2017\_IN\_CTX\_CGE1\_UPREG[GSEA]

|  || Dataset | state43017 |
| Phenotype | NoPhenotypeAvailable |
| Upregulated in class | na\_neg |
| GeneSet | RICHARDS\_NOWAKOWSKI\_SCIENCE\_2017\_IN\_CTX\_CGE1\_UPREG |
| Enrichment Score (ES) | -0.41021574 |
| Normalized Enrichment Score (NES) | -2.236729 |
| Nominal p-value | 0.0 |
| FDR q-value | 0.005128816 |
| FWER p-Value | 0.01 |
Table: GSEA Results Summary

  

Fig 1: Enrichment plot: RICHARDS\_NOWAKOWSKI\_SCIENCE\_2017\_IN\_CTX\_CGE1\_UPREG      
 Profile of the Running ES Score & Positions of GeneSet Members on the Rank Ordered List

  

| PROBE | GENE SYMBOL | GENE\_TITLE | RANK IN GENE LIST | RANK METRIC SCORE | RUNNING ES | CORE ENRICHMENT || 1 | GAS2L3 |  |  | 262 | 0.369 | -0.2993 | No |
| 2 | PHTF2 |  |  | 345 | 0.327 | -0.3612 | Yes |
| 3 | PNRC2 |  |  | 364 | 0.321 | -0.3375 | Yes |
| 4 | XPO1 |  |  | 365 | 0.321 | -0.2894 | Yes |
| 5 | PPME1 |  |  | 423 | 0.299 | -0.3217 | Yes |
| 6 | PPP1R9A |  |  | 435 | 0.295 | -0.2924 | Yes |
| 7 | CEP350 |  |  | 455 | 0.291 | -0.2745 | Yes |
| 8 | NUP50 |  |  | 470 | 0.287 | -0.2504 | Yes |
| 9 | APLP1 |  |  | 480 | 0.285 | -0.2199 | Yes |
| 10 | CDH2 |  |  | 558 | 0.271 | -0.2835 | Yes |
| 11 | RTN1 |  |  | 573 | 0.269 | -0.2622 | Yes |
| 12 | ST8SIA5 |  |  | 588 | 0.266 | -0.2413 | Yes |
| 13 | PUM2 |  |  | 613 | 0.261 | -0.2346 | Yes |
| 14 | PCBP2 |  |  | 660 | 0.255 | -0.2587 | Yes |
| 15 | DCX |  |  | 689 | -0.264 | -0.2570 | Yes |
| 16 | PLS3 |  |  | 700 | -0.275 | -0.2293 | Yes |
| 17 | CHD7 |  |  | 706 | -0.284 | -0.1935 | Yes |
| 18 | SOX4 |  |  | 712 | -0.305 | -0.1545 | Yes |
| 19 | BTG1 |  |  | 741 | -0.423 | -0.1290 | Yes |
| 20 | MLLT11 |  |  | 744 | -0.430 | -0.0673 | Yes |
| 21 | SOX11 |  |  | 754 | -0.576 | 0.0068 | Yes |
Table: GSEA details [plain text format]

  

Fig 2: RICHARDS\_NOWAKOWSKI\_SCIENCE\_2017\_IN\_CTX\_CGE1\_UPREG: Random ES distribution      
 Gene set null distribution of ES for **RICHARDS\_NOWAKOWSKI\_SCIENCE\_2017\_IN\_CTX\_CGE1\_UPREG**
